# A comparative study of pediatric open pyeloplasty, laparoscopy-assisted extracorporeal pyeloplasty, and robot-assisted laparoscopic pyeloplasty

**DOI:** 10.1371/journal.pone.0175026

**Published:** 2017-04-20

**Authors:** Sang Hoon Song, Chanwoo Lee, Jaeyoon Jung, Sung Jin Kim, Sungchan Park, Hyungkeun Park, Kun Suk Kim

**Affiliations:** 1 Department of Urology, Asan Medical Center, University of Ulsan College of Medicine, Seoul, Korea; 2 Department of Urology, Ulsan University Hospital, University of Ulsan College of Medicine, Ulsan, Korea; UNITED STATES

## Abstract

**Purpose:**

To compare the outcomes of open pyeloplasty (OP), laparoscopy-assisted extracorporeal (LEXP), and robotic-assisted laparoscopic pyeloplasty (RALP) for ureteropelvic junction obstruction in pediatric patients.

**Methods:**

We retrospectively reviewed the age-matched cohort of 30 children who underwent OP, 30 who underwent LEXP, and 10 who underwent RALP at a single institution, from 1996 to 2014. Pre- and post-operative variables including success rate were compared among surgical groups.

**Results:**

The mean age of the patients was 120.2 months, the Society for Fetal Urology grade was 3.6, the anteroposterior diameter was 3.1 cm, and the renal relative function was 44.0%. The distribution of laterality, mean body mass index, and preoperative anteroposterior pelvic diameter on ultrasound did not differ among groups. The mean length of hospital stay was significantly shorter in the RALP group (3.2 days) than in the OP (6.6 days) and LEXP (5.8 days) groups (p<0.001). The duration of analgesics use was shorter in the RALP group (1.1 days) than in the other groups (p<0.001). During the mean follow-up period of 49.0, 20.1, and 16.6 months, the success rate was 96.7%, 89.7%, and 100% in the OP, LEXP, and RALP groups, respectively, although this difference was not statistically different (p = 0.499). In multivariate regression analysis, the presence of crossing vessels was the only factor that decreased the success rate (hazard ratio: 46.09, 95% confidence interval: 2.41–879.6, p = 0.011).

**Conclusions:**

Patients who undergo RALP have a reduced hospital stay and lower use of pain medication; however, there is no difference in the success rates for OP, LP, and RALP surgeries. The presence of crossing vessels is a negative prognostic indicator for surgical outcome regardless of the surgical method.

## Introduction

Open dismembered pyleoplasty (OP), originally described by Anderson and Hynes,[1] is the most commonly performed surgical procedure for the treatment of ureteropelvic junction (UPJ) obstruction, with a long-term success rate exceeding 95%.[2] During the last two decades, however, various minimally invasive surgical techniques such as endopyelotomy, laparoscopy-assisted extracorporeal pyeloplasty (LEXP), and robot-assisted laparoscopic pyeloplasty (RALP) have been developed and popularized in clinical practice. Since the first application of laparoscopic pyeloplasty (LP), which was pioneered by Peters et al.[3], the laparoscopic approach has not been as popular in pediatric urology as in the adult population[4], possibly due to its technical difficulty and long learning curve.

The robotic surgical technique has garnered profound attention because it not only offers the advantages of conventional laparoscopy regarding minimal perioperative morbidity, but it also has a more rapid learning curve and enables increased visualization and enhanced manipulation of tissues.[5, 6] In addition, RALP is superior in terms of decreased hospital stay and analgesic use compared to OP, although its operation times are longer.[7, 8] In a previous meta-analysis that compared laparoscopic and RALP, the latter procedure had shorter operative times without any significant differences regarding perioperative complications, hospital stay, or success rate.[9]

To date, some reports including meta-analysis have demonstrated the efficacy and safety of minimally invasive pyeloplasty compared to OP in the pediatric population.[9–12] However, there have been few studies, especially in Asian countries, directly comparing OP, LP, and RALP surgical techniques. In our present study, we describe the clinical characteristics and compare the surgical outcomes of OP, LEXP, and RALP.

## Materials and methods

We retrospectively reviewed the medical records of patients who underwent pyeloplasty due to UPJ obstruction at our institution between 1996 and 2014. The diagnosis of UPJ obstruction was based on clinical symptoms and imaging studies such as renal ultrasonography (US) and Tc-99m mercaptoacetyltriglycine (MAG3) renal scans. Surgical treatment was indicated when the patient had symptoms such as abdominal or flank pain, progressive hydronephrosis, or renal functional deterioration. Of the patients who underwent pyeloplasty, excluding bilateral UPJ obstruction cases, OP was performed in 180 patients, LEXP in 35 patients, and RALP in 10 patients. The surgical method was chosen according to the surgeons’ preference. After matching the patients for age, 30 children in the OP group, 30 in the LEXP group, and 10 in the RALP group were included in our present study. Perioperative data such as operation time, length of hospital stay, Foley and drain duration, postoperative pain scale (Face, Legs, Activity, Cry, Consolability [FLACC] scale, range of 0–10), and analgesic usage were collected. Postoperative US was performed at 1, 3, 6, and 12 months, and annually thereafter. A Tc-99m MAG3 renal scan was performed at 6 and 12 months, and annually thereafter. A successful surgery was defined as resolution of patient symptoms, improvement of hydronephrosis on follow-up US, and improvement in drainage on diuretic renal scan without ureteral stent re-insertion or repeat pyeloplasty. Statistical analyses were performed using SPSS version 18.0 (IBM Corp., Amonk, NY). Comparisons between groups were performed using the chi-square or Fisher’s exact test for qualitative variables and the Mann-Whitney test for quantitative variables. Binary logistic regression analysis was used in multivariate analysis to identify factors that predicted unfavorable surgical outcomes.

### Technique

For OP, the conventional Anderson-Hynes dismembered pyeloplasty was performed. With the anterior subcostal incision, muscle layers were split until the Gerota fascia is identified. The fascia was incised and the renal pelvis was exposed anteriorly. The surrounding tissue of UPJ was freed from the UPJ. After confirming adequate ureteral length, the ureter was transected at the UPJ. We used absorbable 6–0 or 7–0 polyglactin sutures for interrupted and running pelvo-ureteral anastomosis. Double-pigtail stents were only inserted in complex cases. A Penrose drain was placed adjacent to the repair, and brought through a separate stab wound. A Foley catheter was left in place for 2 or 3 days. The Penrose drain was removed before discharge.

LEXP is a hybrid technique of conventional laparoscopic surgery and extracorporeal hand-sewing anastomosis. Patients were placed in the lateral position with 60 degrees of flank elevation. A horizontal skin incision was made in the midclavicular line, slightly caudal to the level of the umbilicus, and the abdominal muscles and peritoneum were dissected under direct vision (Hasson technique). All of the laparoscopic surgeries were performed with the transperitoneal approach. A 12 mm trocar was inserted intraperitoneally, and the abdomen was insufflated with CO_2_ gas at a pressure of 10–12 mmHg. Two additional 5 mm trocars were inserted in the midclavicular line above and below the umbilicus. After mobilizing the colon, the surrounding structures were freed from the UPJ precisely so that the UPJ was completely exposed. The UPJ was then drawn out of the abdominal cavity through the slightly extended camera port site, and subsequently anastomosed extracorporeally identical to the open technique. Double-pigtail ureteral stents and a closed-suction drain (Jackson-Pratt drain) were inserted in most of the cases. A Foley catheter was left in place for 2 or 3 days, and the drain was removed before discharge.

RALP was performed with the da Vinci S or Si Surgical System robot (Intuitive Surgical) following previously reported techniques.[5] The patient was positioned similarly to the LEXP procedure, and then securely taped to the table to avoid displacement during surgery. Adequate paddings were applied under the patient. An 8.5 or 12 mm camera port was placed at the supra-umbilical area, and positioned at least 8 cm apart from each trocar or equidistant from the renal pelvis and the camera port. An additional 5 or 12 mm assistant port was introduced in most patients at the suprapubic area in the midline below the belt line, and was used for introducing the suture material, suction/irrigation, and internal stent, as well as for drainage. The robotic instruments used were a Maryland dissector, hook cautery, monopolar curved or round tip scissors, and a set of needle drivers. Transmesenteric access to the retroperitoneum by a small incision in the mesenteric peritoneum near the UPJ with little or no bowel manipulation was used whenever possible, regardless of the affected side of the patient. The dilated renal pelvis and proximal ureter were identified and precisely dissected. Only small vessels attached to the UPJ were controlled with cautery and transected. When a larger crossing vessel feeding the lower pole of the kidney was identified, it was carefully dissected to simultaneously relieve compression to the UPJ and not to interrupt the vascular supply to the kidney. A vascular hitch suture was performed if needed. A 2–0 Nylon suture (straight needle) for extracorporeal knot tying was used to effectively expose the renal pelvis. The surgical procedures used for RALP were the same steps as those used for OP.

Pyelotomy was performed using redundant pelvic tissue attached to the UPJ as a handle to avoid any touching of the anastomotic area. An interrupted suture with absorbable 5–0 or 6–0 polyglactin and a running suture with 5–0 polyglyconate were used for anastomosis. Antegrade double pigtail catheter placement was performed via the assistant port site with guidewire. When the assistant port was not placed, a 16-gauge angiocatheter was passed through the anterior abdominal wall near the hitch stitch. The stent preloaded with a guidewire was passed through the angiocatheter and into the ureter. To confirm its position, the bladder was filled with saline mixed with indigo carmine. Anterior wall anastomosis was completed after stent positioning. The stent was removed 4 to 6 weeks postoperatively in an outpatient clinic.

## Results

The mean age of the 70 patients was 120.2 months, the Society for Fetal Urology (SFU) grade was 3.6, the anteroposterior diameter was 3.1 cm, and the relative renal function was 44.0%. The patient demographics, preoperative clinical features, peri- and post-operative outcomes are presented in Table 1. Distribution of laterality, mean body mass index, and the preoperative anteroposterior pelvic diameter on US did not differ among the groups. Flank or abdominal pain was the most frequent initial presentation in each of the three groups, and the distribution of initial symptoms did not differ among the groups.

**Table 1:** Patient demographics according to the surgical method used.

	Group 1 (n = 30) OP	Group 2 (n = 30) LEXP	Group 3 (n = 10) RALP	P-value
Age at surgery, med (range), (years)	8.5 (2–19)	10.5 (2–16)	11.0 (4–18)	0.175^a^
Height at surgery, med (range), (cm)	131.1 (87.9–181.3)	146.0 (88.7–180.0)	148.6 (106.3–183.1)	0.083 ^a^
Weight at surgery, med (range), (cm) (kg)	27.0 (11.2–93.6)	39.5 (13.4–75.0)	40.1 (14.5–92.8)	0.240 ^a^
BMI at surgery, med (range) (kg/m^2^)	16.7 (13.9–28.4)	17.6 (13.1–23.1)	17.8 (12.8–28.2)	0.810 ^a^
Gender (Male: Female)	22:8 (73.3:26.7)	20:10 (66.7:33.3)	8:2(80.0:20.0)	0.819
Laterality (Right: Left)	8:22 (26.7:73.3)	6:24 (20.0:80.0)	3:7 (30.0:71.0)	0.647
s-Creatinine at surgery, med (range), (mg/dl)	0.6 (0.3–1.0)	0.6 (0.4–1.4)	0.6 (0.4–1.4)	0.315 ^a^
SFU grade at surgery, med (range)	4.0 (2–5)	3.0 (2–5)	4.0 (3–4)	0.066 ^a^
APPD at surgery, med (range), (cm)	2.8 (1.2–5.8)	3.6 (0.7–7.9)	2.2 (1.2–5.5)	0.488 ^a^
Split renal function at surgery, med (range), (%)	45.9 (13.4–100)	47.4 (23.3–72.0)	43.3 (11.2–52.1)	0.290 ^a^
No. presentation (%)				0.438
• Prenatally detected	5 (16.7)	4 (13.3)	0 (0)	
• Flank pain	18 (60.0)	18 (60.0)	9 (90.0)	
• Incidentally detected	3 (0.0)	3 (10.0)	0 (0)	
• Abdominal mass	0 (0)	1 (3.3)	0 (0)	
• Gross hematuria	1 (3.3)	4 (13.3)	1 (10.0)	
• UTI	3 (10.0)	0 (0)	0 (0)	

SD = standard deviation; OP = open pyeloplasty; LEXP = laparoscopy-assisted extracorporeal pyeloplasty; RALP = robot-assisted laparoscopic pyeloplasty; BMI = body mass index; s-creatinine = serum creatinine; SFU = society of fetal urology; APPD = anteroposterior pelvic diameter; UTI = urinary tract infection;

^a^ = Krusak-Wallis test

Peri- and post-operative outcomes are indicated in Table 2. Fibroepithelial polyps causing UPJO were diagnosed in one and two patients in the OP and LEXP group, respectively. When we dichotomized the etiology into intrinsic (primary and polyps) or extrinsic obstruction, the latter was more frequently observed in the RALP (50.0%) than OP (10.0%) or LEXP groups (23.3%) (*P* = 0.031, Fisher’s exact test). The transmesenteric approach was only attempted in the RALP group, and was feasible in one of three cases (33.3%) of right UPJ obstruction, and five of seven cases (71.4%) of left UPJ obstruction. The operation time was significantly longer in the RALP (254.1 min) than in the OP (192.5 min) and LEXP groups (197.4 min). An intraoperative technical complication occurred in one case in which one finger of the 5 mm needle driver was broken during the procedure when we forcibly grasped a 2–0 straight needle for the hitch stitch. A piece of the needle driver finger was found on the dome of the liver by laparoscopic endoscopy using a C-arm guided intra-operative X-ray.

**Table 2:** Peri- and post-operative outcomes according to the surgical technique.

	Group 1 (n = 30) OP	Group 2 (n = 30) LEXP	Group 3 (n = 10) RALP	*P* value
Etiology				0.622
• Intrinsic-primary	25 (83.3)	21 (70.0)	5 (50.0)	
• Intrinsic-polyp	2 (6.6)	2 (6.7)	0 (0)	
• Extrinsic (crossing vessel)	3 (10.0)	7 (23.3)	5 (50.0)	
No. transmesenteric approach (%)	0 (0)	0 (0)	6 (60.0)	< 0.001
Mean ± SD operation time (mins)	192.5 ± 67.1	197.4 ± 38.9	254.1 ± 46.0	0.008
No. stent insertion (%)	10 (33.3%)	22 (73.3%)	8 (80%)	< 0.001
Mean ± SD Foley duration (days)	3.4 ± 0.8	3.1 ± 0.9	1.7 ± 0.8	< 0.001
Mean ± SD LOS (days)	6.6 ± 1.5	5.8 ± 1.4	3.2 ± 1.0	< 0.001
Mean ± SD analgesic usage duration (days)	3.2 ± 1.0	2.4 ± 1.3	1.1 ±1.2	< 0.001
Complications	4 (13.3)	4 (13.3)	1 (10.0)	0.664
• Intraoperative	0 (0)	0 (0)	1 (10.0)	
• Immediate				
1. Ileus	0 (0)	1 (3.3)	0 (0)	
2. Wound dehiscence	1 (3.3)	0 (0)	0 (0)	
3. UPJ obstruction	1 (3.3)	2 (6.6)	0 (0)	
• Late				
1. Nonfunctioning kidney	0 (0)	0 (0)	0 (0)	
2. UTI	2 (6.6)	1 (3.3)	0 (0)	
Mean ± SD FU duration (months)	49.0 ± 31.8	20.1 ± 15.1	16.6 ± 10.3	< 0.001
No. postop hydronephrosis SFU grade (%)^a^				0.406
• Grade 0	1 (3.3)	3 (10.0)	2 (20.0)	
• Grade 1	9 (30.0)	8 (26.7)	3 (30.0)	
• Grade 2	12 (40.0)	11 (36.7)	1 (10.0)	
• Grade 3	7 (23.3)	7 (23.3)	4 (40.0)	
• Grade 4	1 (3.3)	1 (3.3)	0 (0.0)	
Mean ± SD postop APPD (cm) ^a^	1.5 ± 0.9	1.3 ± 1.0	1.3 ± 0.6	0.667
• % reduction from preop APPD	52.4 ± 31.4	43.2 ± 35.5	47.6 ± 24.5	0.577
Mean ± SD postop split renal function (%)	45.9 ± 10.9	47.2 ± 10.4	34.1 ± 18.0	0.031
• Change from preop function	1.9 ± 4.6	2.6 ± 3.7	3.0 ± 5.7	0.746
Mean ± SD postop T_1/2_ on MAG3 renal scan	6.9 ± 3.7	7.8 ± 4.5	6.8 ± 3.4	0.409

SD = standard deviation; OP = open pyeloplasty; LEXP = laparoscopy-assisted extracorporeal pyeloplasty; RALP = robot-assisted laparoscopic pyeloplasty; LOS = length of stay; UTI = urinary tract infection; FU = follow up; postop = postoperative; preop = preoperative; APPD = anteroposterior pelvic diameter;

^a^ Postoperative hydronephrosis grade and APPD were data collected at the last visit to the clinic

The mean length of hospital stay was significantly shorter in the RALP (3.2 days) than in the OP (6.6 days) and LEXP groups (5.8 days) (*P* < 0.001). The mean time to Foley catheter removal was also significantly shorter in the RALP (1.7 days) than in the OP (3.4 days) and LEXP groups (3.1 days) (*P* < 0.001). Similarly, the duration of analgesic use was shorter in the RALP (1.1 days) than in the other groups (*P* < 0.001). During the mean follow-up period of 49.0, 20.1, and 16.6 months, the success rate was 96.7%, 89.7%, and 100% in the OP, LEXP, and RALP groups, respectively (*P* = 0.499). In multivariate logistic regression analysis, the presence of aberrant vessels was the only factor that decreased the success rate (hazard ratio: 46.09, 95% confidence interval: 2.41–879.6, *P* = 0.011) (Table 3).

**Table 3:** Logistic regression analysis for the prediction of success.

	Univariate		Multivariate		
	OR	*P*	OR	95% CI	*P*
Gender	0.362	328	2.671	0.169–42.240	0.485
Age at op	1.137	0.310	1.018	0.989–1.048	0.233
Op method OP LEXP RALP	Ref 0.299 5.571	0.308 0.999	0.347	0.029–4.210	0.347
Preop HN grade	1.395	0.622			
Preop APPD	1.285	0.561			
Preop split renal function	0.979	0.523			
Dismember or not	0.148	0.140			
Preop stone	1.134	0.999			
Preop UTI	0.254	0.271			
Preop GHU	1.095	0.999			
Preop PCN	1.584	0.999			
Crossing vessel	0.077	0.032	0.077	0.007–0.805	0.032
Stent insertion	0.487	0.543	0.813	0.021–31.834	0.912
PCN insertion	1.095	0.999			

OP = open pyeloplasty; LEXP = laparoscopy-assisted extracorporeal pyeloplasty; RALP = robot-assisted laparoscopic pyeloplasty; HN = hydronphrosis; APPD = anteroposterior pelvic diameter; UTI = urinary tract infection; GHU = gross hematuria; PCN = percutaneous nephrostomy

Among the four patients who failed to recover renal function after the first pyeloplasty, three had crossing vessels. A 13-year-old female patient had a renal artery that crossed the lower moiety UPJ of the left duplicated kidney, and could not be transposed during the LEXP. She underwent secondary endoscopic balloon dilation and tertiary endopyelotomy, resulting in preserved renal function and disappearance of symptoms. An 8-year-old male had an intraoperatively detected lower pole artery and a vein crossing the UPJ, which was not observed by preoperative computed tomography urography and was not transposed during LEXP. The patient underwent subsequent OP re-do due to persistent symptoms 1 month after the first operation, which led to stabilization with preserved renal function. The third patient with crossing vessels was a 6-year-old female with horseshoe kidney and UPJ obstruction on the left side. The crossing vessel was hitched during the OP without posterior transposition to the ureter. She underwent percutaneous nephrostomy (PCN) insertion and antegrade balloon dilatation due to recurrence of symptoms right after removal of the double J stent after the first surgery. Another patient without crossing vessels underwent successful OP re-do after failure of LEXP due to anastomosis leakage and urinoma collection.

**Fig 1:**
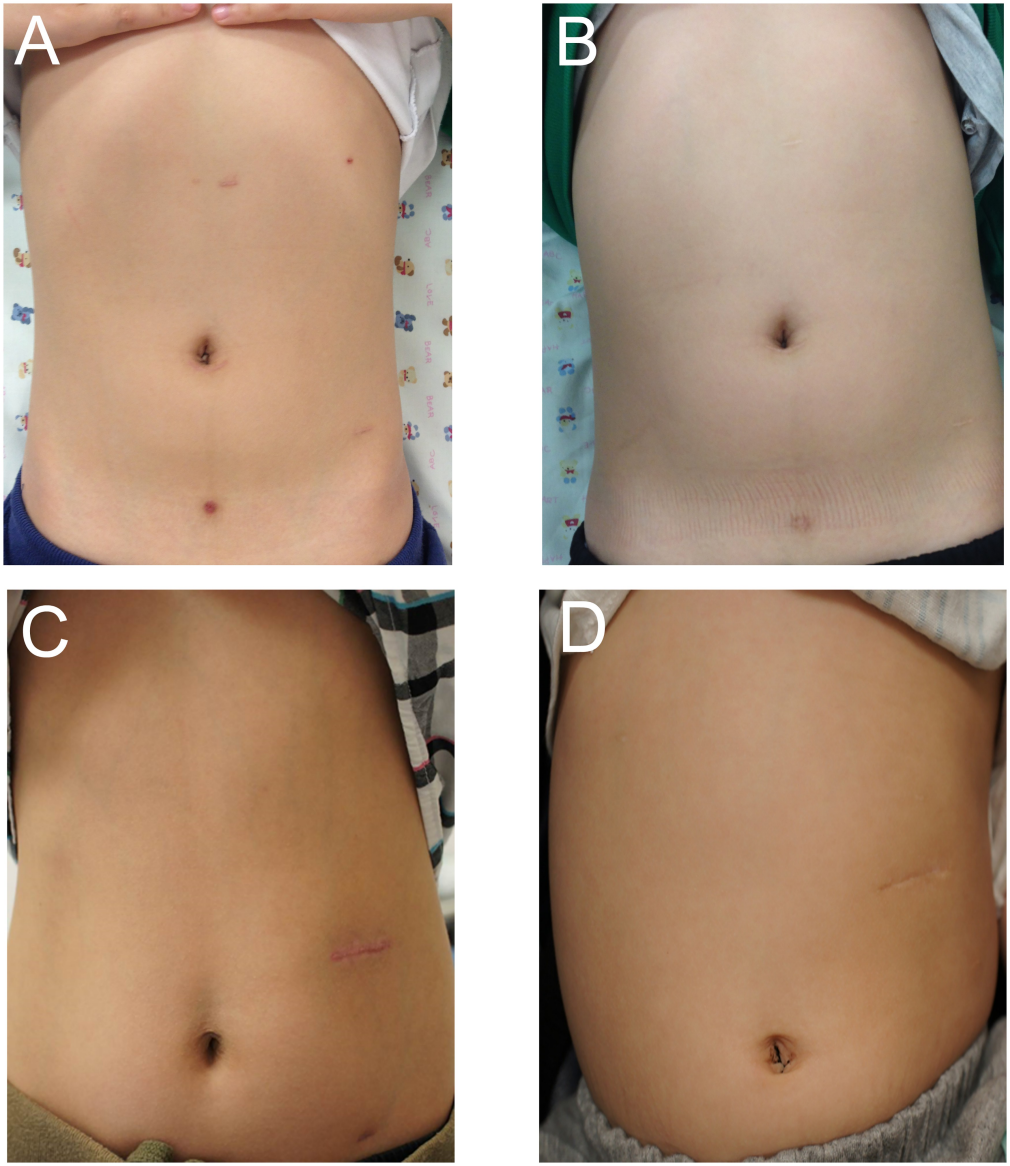
Cosmetic outcome. Cosmetic outcome after robot assisted laparoscopic pyeloplasty (RALP) was superior than that for laparoscopy-assisted extracorporeal pyeloplasty (LEXP) and open pyeloplasty (OP). For RALP, port-site scars were seen 1 month after operation (A) and became much fainter 1 year after the operation (B). The size of the abdominal scar was shorter with LEXP (C) than with OP (D), although an additional port-site scar was seen in the left lower quadrant in patients who underwent LEXP (C).

Favorable cosmetic results were observed after RALP (Fig 1). For robot RALP, 5 mm port-site scars were very faint at 1 year after operation. The size of the upper abdominal scar was slightly smaller after LEXP compared to OP.

## Discussion

To the best of our knowledge, our current study is the first to compare OP, LP, and RALP in a single-center study. Our patients who underwent RALP not only had a decreased hospital stay and lower use of pain medication compared to those who underwent OP or LEXP, but this procedure also had a higher success rate, which is in accordance with data from previous studies.[7, 8] This finding also concurs with a previous meta-analysis, which showed that minimally invasive pyeloplasty and OP perform equally in terms of success rate.[9]

In contrast to the results from the aforementioned meta-analysis, however, the length of hospital stay in our study was significantly different between the OP and minimally invasive surgery groups. Specifically, the mean hospital stay in the previous meta-analysis cohort was 3.5 days in the minimally invasive group and 4.3 days in the OP group, resulting in a -0.86 mean difference. However, in our study series, the RALP group had a shorter hospital stay (3.2 days) than the OP group in the pooled cohort of participants in the previous meta-analysis, and the OP (6.3 days) and LEXP (5.8 days) groups had longer hospital stays than the cohort who underwent minimal invasive surgery (4.3 days). The hospital course could have been extended in patients who underwent LEXP because of the surgical technique regarding colon mobilization compared to those who underwent RALP. However, even the four patients who underwent colonic mobilization in the RALP group had a similar length of hospital stay (2.7 days vs 3.5 days, p = 0.286) and pain medication duration (1.0 day vs 1.1 day, p = 0.812) with the six patients who underwent the transmesenteric approach. Moreover, the duration of analgesic use was shorter in the RALP group than in the other groups because of the smallest skin incision and the avoidance of subcostal incision. The difference in length of hospital stay cannot be ignored considering that a child’s hospital care represents a significant burden to working parents, as reported by Behan et al.[13] that RALP is associated with human capital gains and lower hospitalization expenses. After setting aside the distinguishing feature of flexible hospital stay due to the low cost (about 7.4 US dollars per day[14]) of hospitalization in Korea, there is economic value to parents in reducing pain and emotional distress even after discharge, because children need prolonged care by one or two parents until they gain a comfortable level of recovery, which was not measured or estimated in our present retrospective analysis.

**Fig 2:**
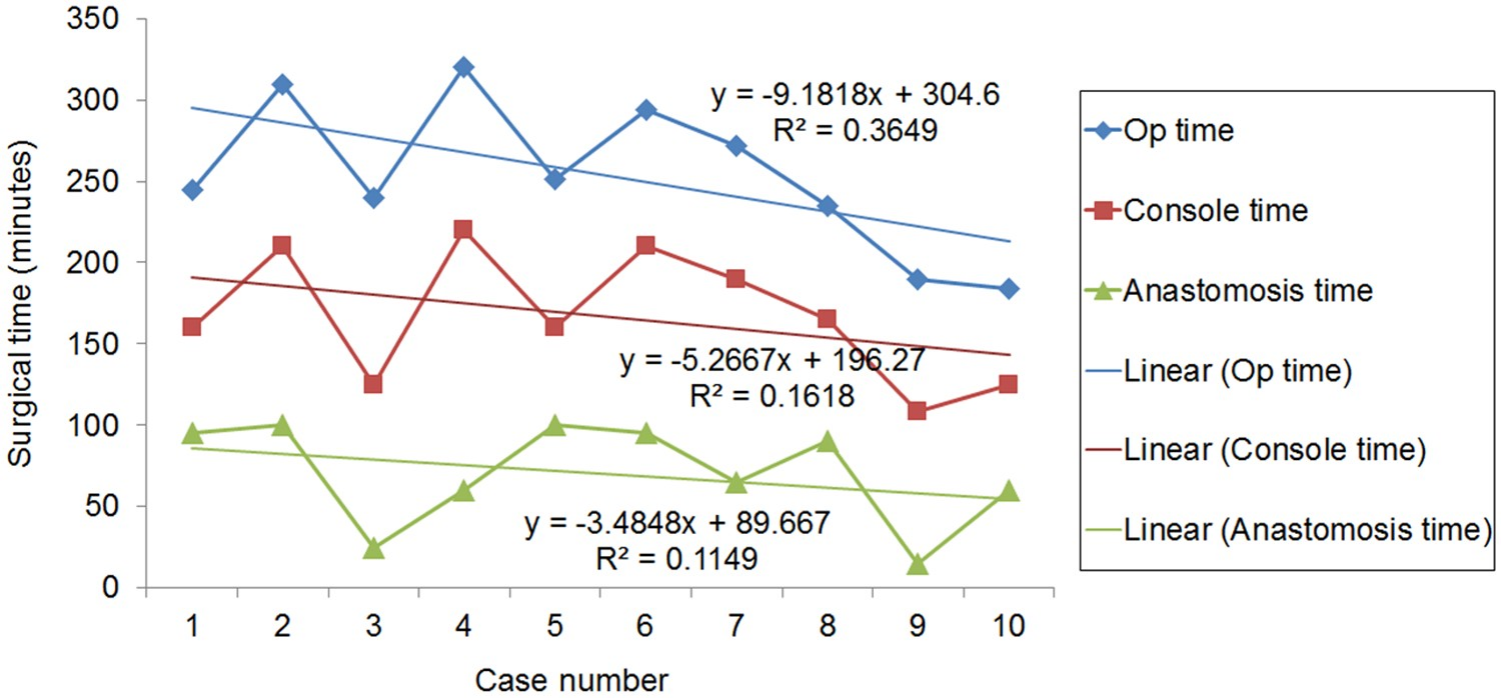
Learning curve of robot assisted laparoscopic pyeloplasty. Linear graph of perioperative time, console time, and anastomosis time as a function of increasing surgical experience. The total operation time showed a tendency to decrease with the surgeon’s experience with robot assisted laparoscopic pyeloplasty cases (*P* = 0.061).

According to previous studies comparing RALP and LP in children, RALP is associated with a shorter operative time.[15, 16] However, in our present study, the operation time was significantly longer in the RALP group, partly due to the additional time needed for robot docking, and partly because the 10 RALP cases in our study series were the first to receive this treatment at our institution, so there was a learning curve period. However, the total operation time, console time, and anastomosis time tended to decrease with increasing experience (Fig 2). The operating times for OP and LEXP did not significantly differ because both surgeries were performed by handsewing the anastomosis. Nonetheless, before the introducction of RALP, LEXP was the preferred choice of surgery in our institution for school-aged and adolescent patients due to the advantage of being able to decrease the upper abdominal scan to about 2–3 cm. On the other hand, it is more technically demanding to locate the UPJ site in OP.

Extrinsic obstruction by crossing vessels over the UPJ was more common in RALP than in the other procedures. Moreover, in multivariate logistic regression analysis, the presence of an aberrant vessel was the only factor that decreased the success rate. Lucas et al. reported that crossing vessels identified intraoperatively was a significant predictor of reduced freedom from secondary procedures.[17] These authors found crossing vessels in 46.5% of patients who underwent RALP, similar to our results (50.0% in RALP), and showed that this resulted in a higher subjective failure rate (5.5% vs. 2.2%) and a higher rate of secondary procedures (8.5% vs. 2.2%). In our present analysis, the failure rate was also significantly higher in patients with crossing vessels (20.0% vs. 1.9%), although failed cases were only identified in the OP (33.3%) and LEXP groups (28.6%) groups (*P* = 0.385). Likewise, a 100% success rate was reported previously with minimally invasive pyeloplasty in patients with lower pole crossing vessels.[18, 19] However, the role of crossing vessels in the management of UPJ obstruction remains unclear. Anterior anastomosis of the ureter and renal pelvis over the crossing vessel is sometimes technically demanding due to the concern regarding tension-free anastomosis.[19] It seems reasonable that the decision to transpose the ureter should be made by subjective intraoperative assessment according to the surgeon’s judgment.[18] Nevertheless, we postulate that the underestimation or improper management of crossing vessels, especially in the OP and LEXP groups, could be the cause of failed surgery, as this procedure demands a meticulous technique and precise judgment of the surgeon. The satisfactory outcomes from RALP in our present study despite the 50% incidence of crossing vessels may be evidence of the value of this surgery, which allows fastidious inspection with a magnified view of the UPJ by 3D vision, and dexterity to reconstruct the urinary tract with deliberation.

The mean age of the patients in our present study series was above 11 years because the RALP group had the lowest number of patients with a mean age older than that in the OP group, resulting in an overall increase in the mean age of the study population compared to that in previous studies comparing OP and RALP.[8, 10, 11] However, the age of patients who undergo minimally invasive pyeloplasty can be even lower. It has been reported that LP is the standard treatment for UPJ obstruction in children from 3 years of age[20], and even from 4 months of age[21] in some institutions. Nevertheless, pediatric laparoscopic surgery has not been widely adopted, possibly due to its technical difficulty and long learning curve, whereas RALP is the most commonly reported robotic surgery in children.[22] Dangle et al. reported that the success rate of RALP was comparable to OP, even in infants.[23]

In terms of limitations, our present study involved a retrospective cohort. In addition, due to the order of introduction of surgical technique from OP to LEXP, and then to RALP, patients in each surgical group reflected a chronological order so that the surgeon’s experience might have played a role in the outcome of the surgery which could not be compensated for by the statistical method we used. Furthermore, the mean age of the patients were more than 120 months, so that it could be limited in application of our experience to the infants and toddlers. The sample size of our study was also small, so a follow-up study with a larger cohort is warranted. In addition, the LEXP group in our study might not be the representative group for patients who undergo laparoscopic pyeloplasty because the anastomosis was sawn by hand, not by laparoscopic instrument.

## Conclusions

Patients who underwent RALP have a decreased length of hospital stay and lower use of pain medication. However, there is no difference in the success rate between OP, LEXP, and RALP. The presence of crossing vessels is a predictive factor for poor surgical outcome regardless of the surgical method. The satisfactory results with RALP, despite the high incidence of crossing vessels, may be evidence of the value of this surgery, which allows for a precise inspection of the UPJ area and delicate reconstruction of the urinary tract.
